# Road traffic injuries measures in the Eastern Mediterranean Region: findings from the Global Status Report on Road Safety – 2015

**DOI:** 10.5249/jivr.v11i2.1122

**Published:** 2019-07

**Authors:** Soori Hamid, Khorasani-Zavareh Davoud

**Affiliations:** ^*a*^Safety Promotion and Injury Prevention Research Center, Shahid Beheshti University of Medical Sciences, Tehran, Iran.; ^*b*^Department of Health in Emergencies and Disasters, School of Public Health and Safety, Shahid Beheshti University of Medical Sciences, Tehran, Iran.; ^*c*^Department of Neurobiology, Care Sciences and Society (NVS), H1, Division of Family Medicine and Primary Care, Alfred Nobels Allé 23 141 83 Huddinge, Stockholm, Sweden.

**Keywords:** Road safety, Epidemiology, Preventive, Measures

## Abstract

**Background:**

The Eastern Mediterranean Region has the second highest road traffic fatality rate in the world. This article presents the epidemiology of road traffic injuries and the preventive measures in Eastern Mediterranean Region taken by the different World Health Organization member states compared to the rest of the world.

**Methods:**

This is a secondary data analysis addressing the Global Status Report on Road Safety published by the World Health Organization in 2015. Data are from 180 countries covering 6.97 billion people of the world’s population, of which 21 Eastern Mediterranean Region of World Health Organization member states with about 595 million population were included and were analyzed. From 22 countries in the region, 21 are presented and Syria has not reported any data on road traffic injuries.

**Results:**

Eastern Mediterranean Region member states contribute to 9.69% of all global fatal road traffic injuries (19.9 per 100 000 population compared to the same rate in the European region with 9.3), while these countries account for 7.4% of the world’s population and have about 5.6% of the world’s vehicles on their roads. More than 90% of the Eastern Mediterranean Region countries have passed mandatory seat-belt laws for both front-seat and rear-seat passengers and making helmet use obligatory; and only 27% have child restraint laws; half percent have an emergency room injury surveillance system. All countries have a national drink-driving law; and certain speed limits but there is no distinction between rural and urban areas, and the latter lack adequate speed restrictions.

**Conclusions:**

Although the Eastern Mediterranean Region member states have some important preventive measures recommended by World Health Organization, considerable efforts are still needed to optimize the enforcement of existing road safety laws. The maximum urban speed limit should be reduced in many countries. Sufficient attention should be paid to the needs of pedestrians, cyclists and motorcyclists, who together make up about 50% of Eastern Mediterranean Region road traffic deaths.

## Introduction

Road traffic injuries (RTIs) are a major global public health problem^1,2^ and the Eastern Mediterranean Region (EMR) of the World Health Organization (WHO) ranked second in the world report on road traffic injury prevention.^3-5^The first report on the subject was developed jointly by WHO and the World Bank and presents the current knowledge about RTIs and what can be done to address the problem.^2^The Decade of Action for Road Safety (2011–2020) calls on countries to implement the measures identified internationally to make their roads safer.^6,7^ The UN General Assembly invited WHO to monitor progress through its Global Status Re-port on road safety series.^1,3,8^This series of reports was published in 2009, 2013 and 2015.^1,3,8^ Having a better understanding on the status of road safety in EMR and comparing the related figures between both EMR states and the global figures can give us a clearer picture and enable us to have more effective policies in the region to achieve the Decade of Action for Road Safety goals.^6,9^

The EMR has the second highest road traffic fatality rate in the world.^1,3,9-11^ There is, however, a significant discrepancy in these figures for the different EMR states.^12^ Whereas high-income countries (HICs) globally have lower rates than low- and middle-income countries (LMICs),^2^in the EMR, the HICs have higher fatality rates than their less-affluent neighbors.^9^

When the WHO launched its call for action, it put emphasis on members of the public being a part of the solution in these following activities.^1,3,8,13^ The initiative focused on five important courses of action for the general public, which included: observing the speed limits; wearing a seat-belt; wearing a motorcycle helmet; never drinking and driving; and being visible on the road. In addition, child restraints and not texting while driving were also added to these courses of action.^1^ They are for the most part already observed in most high-income countries. However, it is important to see how these actions, as well as other preventive measures (see data collection and data analysis procedure in method section) in the EMR region in order to deal with the large number of fatal RTIs. On the other hand, an examination of the epidemiology of RTIs is one of the most important steps for any preventive activities.^14,15^ The present article therefore presents the epidemiology of RTIs and the preventive measures in the EMR region by different EMR states compared to the rest of the world.

**Figure 1:**
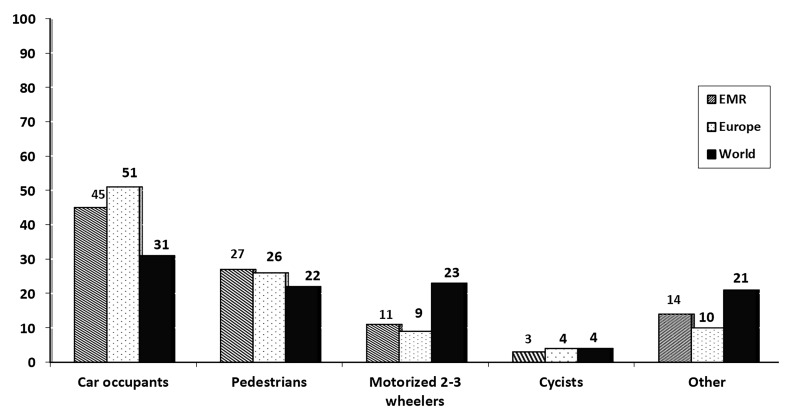
Fatal road traffic injuries by type of road users in Eastern Mediterranean, Europe and world in 2015.

## Methods 

This is a secondary data analysis addressing the Global Status Report on Road Safety published by the WHO in 2015.^1^ This report was the third in the series,^1,3,8^ to assess the state of RTIs in different member countries. Data are from 180 countries/areas covering 6.97 billion people of the world’s population including 21 out of 22 EMR member states with a population of about 595 million (96.5% of total EMR population).^1^ The Eastern Mediterranean countries that participated in this report are Afghanistan, Bahrain, Djibouti, Egypt, Iran, Iraq, Jordan, Kuwait, Lebanon, Libya, Morocco, Oman, Pakistan, Qatar, Saudi Arabia, Somali, Sudan, Tunisia, United Arab Emirates, West Bank and Gaza and Yemen. The data from Syria, which covers 3.5% of total EMR population, have not been reported.^1^

**Data collection and data analysis procedure**

Data were collected by trained national coordinators with the collaboration of up to eight other road safety experts within their country from the different sectors (e.g. health, police, transport, nongovernmental organizations and/or academia). Some other data were based on information from the UN World Forum for Harmonization of Vehicle Regulations,^16^ United Nations Population Division database (Population Division June 2013);^17^ and World Bank (World Development Indicators database).^18^ The World Bank Atlas method was used to categorize Growth National Income (GNI). Death was adjusted for the 30-day definition of a road traffic death^2^ and the modeled number of deaths calculated using negative binomial regression. ^1^

In summary, the work on the report had begun in May 2014 and was completed by December 2014 with the specific objectives of:

• Describing the road safety situation in all Member States;

• Identifying gaps in road safety in all Member States and thereby stimulating road safety action;

• Monitoring countries’ progress in implementing measures identified in the Global Plan of Action for the Decade of Action for Road Safety (2011–2020); and

• Providing baseline information and data that allow monitoring of other international policy processes that set road safety targets.

Variables which were selected from the Global Status Report on Road Safety for this paper are: Countries in EMR; Total population; Number of registered vehicles; Proportion of vehicles per 100 persons; Country reported number of deaths; Modeled number of deaths (WHO report); Estimated road traffic death rate per 100 000 population (WHO estimation); Correction factor of fatal RTIs (estimation number of fatal RTIs by the country compared to estimation rate by WHO); Percentage of fatal road traffic injuries by category of road user; National legislation on speed limit including maximum urban speed limit law; Mandatory seat-belt laws for both front-and rear seat occupants with level of enforcement; Mandatory helmet laws for both drivers and passengers with level of enforcement; Child restraint law; Random breath testing or police check points; National law on mobile phone use while driving; Emergency room injury surveillance system; National road safety strategy; Reported policies to encourage investment in public transport. Data were re-analyzed using Excel 2010. These variables currently are measures and key points for interventions in road safety; of which World Health Organization follow them in all countries. 

## Results

EMR states contribute globally to 9.69% of all RTI fatalities (19.9 per 100 000 population presented in the original report compared to the same rate in the European region with 9.3 per 100 000 population). This figure is 22.4 as calculated by the authors. Overall, the EMR includes a population of about 595 million, which is 7.4% of the total world population. In this report, results from 21 out of 22 countries in the region are presented: Syria has not reported any data on RTIs. Generally, 28.6% of this population live in EMR high-income countries while the rest are in LMICs. As the report indicates, the EMR is the only region where HICs have a higher road traffic death rate than LMICs.

Overall in the EMR, fatal RTIs comprise: 45% car occupants, 27% pedestrians, 11% riders of motorized two- or three -wheelers, 3% cyclists and 14% other road users.

Table 1 shows the frequency and percent of total population and registered vehicles in 21 different EMR states. The total number of registered vehicles in the region is 74 881 962, with 66.2% from Iran, Pakistan, Egypt and Saudi Arabia, which contributes towards only 5.6% of the total registered vehicles globally. More than half of the EMR population (57.4%) live in Pakistan, Egypt and Iran.

**Table 1:** Frequency and percent of total population and registered vehicles in 21 different countries of Eastern Mediterranean Region in 2015.

Country ^a^	Population number (%)	Total Registered Vehicles (%)	Proportion of vehicles per 100 persons
Afghanistan	30551674 (5.1)	655357 (0.9)	2.1
Bahrain	1332171 (0.2)	545155 (0.7)	41
Djibouti	872932 (0.1)	-	-
Egypt	82056378 (14)	7037954 (9.4)	8.5
Iran	77447168 (13.7)	26866457 (35.9)	35
Iraq	33765232 (5.7)	4515041 (6)	13
Jordan	7273799 (1.2)	1263754 (1.7)	17
Kuwait	3368572 (0.6)	1841416 (2.5)	55
Lebanon	4821971 (0.8)	1680011 (2.2)	35
Libya	6201521 (1)	3553497 (4.7)	57
Morocco	33008150 (5.5)	3286421 (4.4)	10
Oman	3632444 (0.6)	1082996 (1.4)	30
Pakistan	182142594 (31)	9080437 (12.1)	5
Qatar	2168673 (0.4)	647878 (0.9)	30
Saudi Arabia	28828870 (4.8)	6599216 (8.8)	23
Somalia	10495583 (1.8)	59457 (0.1)	0.6
Sudan	37964306 (6.4)	320974 (0.4)	0.8
Tunisia	10996515 (1.8)	1735339 (2.3)	16
United Arab Emirates	9346129 (1.6)	2674894 (3.6)	29
West Bank and Gaza	4326295 (0.7)	233818 (0.3)	5.4
Yemen	24407381 (4.1)	1201890 (1.6)	4.9
**Total**	**595008358 (100)**	**74881962 (100)**	**13**

a Data for Syria are not available. - Data not available.

As shows in Table 2, the highest RTI fatality rates per 100 000 population in the region occurred in Libya (73.4) and Iran (32.1). Focusing on an under-estimation of fatal RTI cases, the highest under-estimation rate related to Somalia followed by Afghanistan, while Oman and Saudi Arabia had the best estimation number of fatal RTIs by country compared to estimation rate by WHO.

**Table 2:** Frequency, rate and factor of fatal road traffic injuries (per 100 000 population) in 21 countries of the Eastern Mediterranean Region in 2015.

Country	Population number	Country reported number of deaths ^a^	Modeled number of deaths (WHO report) ^b^	Estimated road traffic death rate per 100 000 population (WHO estimation)^c^	Correction factor ^d^
Afghanistan	30551674	1808	4734	15.5	2.6
Bahrain	1332171	83	107	8	1.2
Djibouti	872932	1030	216	24.7	0.2
Egypt	82056378	8701	10466	12.8	1.2
Iran	77447168	17994	24896	32.1	1.3
Iraq	33765232	5789	6826	20.2	1.1
Jordan	7273799	768	1913	26.3	2.4
Kuwait	3368572	473	629	18.7	1.3
Lebanon	4821971	630	1088	22.6	1.7
Libya	6201521	4398	4554	73.4	1
Morocco	33008150	3832	6870	20.8	1.7
Oman	3632444	913	924	25.4	1
Pakistan	182142594	9917	25781	14.2	2.5
Qatar	2168673	240	330	15.2	1.3
Saudi Arabia	28828870	7661	7898	27.4	1
Somalia	10495583	201	2664	25.4	13
Sudan	37964306	2281	9221	24.3	4
Tunisia	10996515	1505	2679	24.4	1.7
United Arab Emirates	9346129	651	1021	10.9	1.5
West Bank and Gaza	4326295	133	241	5.6	1.8
Yemen	24407381	3239	5248	21.5	1.6
**Total**	**595008358**	**72247**	**118306**	**22.4c**	**1.6**

a Adjusted for 30-day definition of a fatal road traffic injury. b Modeled using negative binomial regression (see http://www.who.int/violence_injury_prevention/road_safety_status/2015/en/ for detailed methodology). c This figure has been reported as 19.9 in the original report. d The correction factor calculated as dividing reported number of death by country to reported number of deaths by WHO.

Table 3 presents fatal road traffic injuries by category of road user in the EMR. Focusing on vulnerable road users, the highest proportion of two- or three-wheel vehicle-riders was in Iran followed by Morocco and Tunisia; while fatalities among pedestrian road users mainly occurred in the West Bank and Gaza followed by Lebanon. However, fatal car accidents mainly occurred in Qatar and Libya.

**Table 3:** Percentage of fatal road traffic injuries by category of road user in the Eastern Mediterranean Region in 2015.

Country^a^	Drivers/occupants of 4-wheeler vehicles	Pedestrians	Cyclists	Drivers/occupants of 2- or 3-wheelers vehicles ^b^	Others
Bahrain	59	31	6	4	-
Egypt	49	29	6	1	15
Iran	41	23	1	22	13
Jordan	64	36	-	-	-
Lebanon	-	43	-	-	57
Libya	71	26	1	2	-
Morocco	36	26	6	21	11
Oman	64	23	3	2	8
Qatar	72	28	-	-	-
Sudan	-	33	-	-	67
Tunisia	49	28	3	21	-
United Arab Emirates	57	26	1	3	14
West Bank and Gaza	54	44		2	-

a Data for Afghanistan, Djibouti, Iraq, Kuwait, Pakistan, Saudi Arabia, Somalia, and Yemen not available. b Including light vehicles.

In 21 countries in the region, the national speed limits (km/h) in urban areas for vehicles ranged from 45 to 100. Apart from Afghanistan and Somalia that have no law for seat-belt use, helmet wearing or a national law on mobile phone use while driving; all countries have mandatory laws for such behavior with a level of enforcement ranged from 2 till 10. Only around 50% have child restraint laws; and close to 50% have emergency room injury surveillance system. Fifteen countries have policies to encourage investment in public transport. More details about measures on laws and legislations in the different countries of EMR are presented in Table 4. Low enforcement scores (overall 5 out of 10) in most EMR countries contribute to a low or non-effective role on road safety laws to reduce injuries and deaths.

**Table 4:** Some measures on laws and legislations in different countries of Eastern Mediterranean Region in 2015.

Countries ^a^	Max urban speed limit law	Mandatory seat-belt laws for both front-and rear seat occupants (level of enforcement)^b^	Mandatory helmet laws for both drivers and passengers (level of enforcement)^b^	Child restraint law (level of enforcement)^b^	Random breath testing or Police check points	National law on mobile phone use while driving	Emergency room injury surveillance system	National road safety strategy	Policies to encourage investment in public transport
Afghanistan	No	No (-)	No (-)	No (-)	No	No	No	No	No
Bahrain	60	Yes (7)	Yes (9)	Yes (0)	No	Yes	No	Yes	Yes
Djibouti	50	Yes (3)	Yes (4)	No (-)	Yes	No	No	No	No
Egypt	60	Yes (8)	Yes (5)	No (-)	Yes	Yes	Yes	Yes	No
Iran	60	Yes (7)	Yes (5)	No (-)	Yes	Yes	Yes	Yes	Yes
Iraq	60	Yes (5)	Yes (2)	No (-)	No	Yes	Yes	Yes	Yes
Jordan	90	Yes (6)	Yes (4)	No (-)	Yes	Yes	No	Multiple	Yes
Kuwait	45	Yes (3)	Yes (7)	No (-)	Yes	Yes	No	Yes	Yes
Lebanon	50	Yes (3)	Yes (2)	Yes (0)	Yes	Yes	No	Yes	No
Libya	50	Yes (3)	Yes (1)	No (-)	No	Yes	No	Yes	Yes
Morocco	60	Yes (7)	Yes (8)	No (-)	-	Yes	No	Yes	Yes
Oman	N/A	Yes (9)	Yes (10)	Yes (5)	Yes	Yes	Yes	Yes	Yes
Pakistan	90	Yes (3)	Yes (2)	No (-)	Yes	Yes	No	No	Sub-national
Qatar	100	Yes (7)	Yes (8)	No (-)	No	Yes	Yes	Yes	Yes
Saudi Arabia	80	Yes (5)	Yes (3)	Yes (2)	No	Yes	Yes	Yes	Yes
Somalia	40	No (-)	No (-)	No (-)	No	No	Yes	No	No
Sudan	50	Yes (8)	Yes (5)	No (-)	No	Yes	No	Yes	Sub- national
Tunisia	50	Yes (2)	Yes (3)	No (-)	Yes	Yes	No	Yes	Yes
United Arab Emirates	90	Yes (10)	Yes (10)	No (-)	Yes	Yes	Yes	Yes	Yes
West Bank and Gaza	50	Yes (7)	Yes (6)	Yes (6)	No	Yes	Yes	Yes	No
Yemen	No	Yes (2)	Yes (2)	No (-)	No	Yes	Yes	Yes	Yes

a Alcohol consumption is legally prohibited. a Data for Syria are not available. a All EMR states in this report have a national drink–driving law and a national drug-driving law with a level of enforcement ranging from 1 to 10. b The numbers are level of enforcement.

## Discussion

This study found that about 9.7% of global deaths from RTIs occur in the EMR. While all EMR countries have a national drink–driving law, only half of them have random breath testing or police check points to monitor this. Apart from Somalia and Afghanistan, all the other EMR countries have mandatory seat-belt laws for both front-seat and rear-seat passengers. Only one fourth of EMR countries have child restraint laws, and this needs more attention in highly populated countries such as Iran, Pakistan, and Egypt that have no such law. The focus on observing the speed limit is not great in the EMR region. In addition, national speed limits in urban areas for vehicles range from 45 to 100 km/h. Overall, there is 1.6 under-estimated road traffic death rate per 100 000 population (comparison by WHO estimation and country estimation), of which the best estimation of the number of fatal RTIs was related to Saudi Arabia and Oman followed by Iraq.

Overall, in the EMR region fatal RTIs comprise 45% car occupants, 27% pedestrians, 11% riders of motorized two- and three-wheelers, 3% cyclists and 14% other road users.^3^ This figure is 31%, 22%, 23%, 4% and 21% in the world, respectively.^3^ The figure for road user fatalities differs between the EMR and the rest of the world, particularly among car occupants and motorized two- and three-wheelers. While in the EMR states, the proportion of fatality for car occupants is 51%, this figure is 31% globally. This is not something that is expected in countries such as these, as close to two thirds of them are LMIC. More exploration is needed to explain this finding. Fatalities among vulnerable road users are dominant in EMR countries. The reason for that may be partly related to lack of attention to the needs of vulnerable road users when building infrastructure in such regions.^19,20^ Other reasons also are related to mixed road conditions for both vulnerable and protected road users in most EMR states.^21^ Moreover, 9% of fatal RTIs are motorized two- and three-wheelers in EMR while this figure is around 23% globally. The study showed that low-income countries usually suffer from a high rate of fatalities among vulnerable road users like motorized two- and three- wheelers, which is as expected in predictions for such countries.^2,22^ The same explanations can be raised as above, since there is a lack of infrastructure for motorized two- and three-wheelers in most parts of EMR.^23^ By contrast, in most HICs the majority of activities are focused on the safety of vulnerable road users.^24,25^ Oman and Qatar had the highest proportion of fatalities among car passengers in the EMR. The reason for this can be related to the structure of these two countries, which presupposes that the most common mode of transportation will be vehicles rather than walking. For example, Iran, Morocco and Tunisia had the highest proportion of riders of motorized two- and three-wheelers. On the other hand, the West Bank and Gaza and Lebanon had the highest proportion of pedestrian fatalities, which is in line with many studies, which show that the statistics for fatal RTIs in low-income countries are dominated by vulnerable road users. Spatial and non-spatial determinants and analysis can help to provide better pictures and policy making on the phenomena of interest, as other studies stated on its importance for prevention.^26^

In a comparison between the reported number of fatal RTIs by World Health Organization and the number reported by the county, the lowest under-estimation rate occurred with Saudi Arabia and Oman. The reason for that is mainly related to the establishment of trauma registry and injury surveillance in Saudi Arabia many years ago.^27^ Moreover, both countries also have an emergency room injury surveillance system. By contrast, Somalia had the highest under-estimation of fatal RTIs due to considerable under-reporting of fatal road traffic injury. The high correction factor is mainly related to a lack of establishment of a trauma registry as well as an injury surveillance system, which is in line with a previous study in Pakistan with an estimation of a correction factor of 2.59, which resulted in 44% under-reporting of fatal RTIs.^28^ Some studies have already showed that data sources in LMICs mainly suffer from under-estimation of the cases.^29-33^ An additional reason may be related to misclassification in the regular death reports from official sources to the authorities^34^ as well as the challenges implicit in the 30-day definition of a road traffic death.^35^ There is also another reason, namely the lack of attention to reporting fatal RTIs in such countries and a failure to realize its importance, which is in line with a previous study in the region.^29-32,34^

In the EMR, highly populated countries such as Iran, Pakistan, and Egypt still have no child restraint law. The use and effectiveness of such a law have been already demonstrated in many studies for RTI prevention among children.^36-39^ As an example of EMR countries, a study in Iran indicated that the reasons for low usage of child restraint are: the high price of a car seat; lack of both knowledge and positive attitude towards them among the public; a lack of willingness to use the car seat by children; and a lack of availability of such protective equipment, particularly the booster seat on the Iranian market.^38^ Studies also indicated that most members of the public don’t know when and how they should use a car seat.^40^ In order to improve the overall usage of the child restraint, providing a loan to help purchase, increasing the availability of the car seat in stores as well as a public education campaign can improve this important measure.^38^ This is also important to improve perception of client, ^19,41^ here, road users and car owner for better coverage of child restraint.

While in most EMR countries a mixture of motorized traffic with pedestrians, cyclists, and motorcycle riders is usual, none of them have an urban 30 km/h speed limit. The focus on speed management is minimal in the EMR region and surprisingly there is no maximum speed limit in Afghanistan, and no enforcement in some countries like Iraq. This goes against findings in successful countries, which focus on speed management in both rural and urban areas. It is important to note that in EMR countries most focus on the speed limit enforcement is on inter-city roads and there is little or no focus on driving speeds in inner-city areas. This means that the large urban populations in such countries are offered no protection from speed management. In situations where motorized traffic mixes with pedestrians, cyclists and moped riders, as is common in most EMR countries, the speed limit isn’t usually under 30 km/h. Based on a system approach, if the country cannot improve the transport system, the best strategy would be to put emphasis on speed management.^42^ However, the findings show that speed management strategies are mainly ignored in LMICs. For instance, rather surprisingly, Afghanistan, Qatar and Yemen have no compulsory speed limits whatsoever. In order to reduce fatal RTIs in such countries, one of the most important strategies, particularly for those that cannot improve the transport system and vehicle system, should be speed management with focus on both inner and inter-city roads.

Almost all countries in EMR have national drink-driving laws and in many of them alcohol consumption is legally prohibited, however, less than half of them have random breath testing or police check points. While mandatory seat-belt laws for both front-and rear seat occupants are in place in almost in all the EMR region, most focus is on front-seat occupants and particularly on drivers and there is low enforcement of rear-seat occupants. This is an important point that should be considered to encourage seat-belt use for all car occupants. Studies have already showed that despite a law and enforcement of seat-belt use, installing seat-belt reminder systems (SBRs) in vehicles is a more effective method for better coverage of seat-belt use in the society.^43^ This means that instead of only focusing on public education and police enforcement, environmental modification should be taken into account for better coverage of seat-belt use in most EMR countries.

Overall, the lack of a system approach is a major challenge in many LMICs and EMR countries; however, this is the foundation for success for RTI prevention in HICs. In successful countries, focus in a system manner is put on members of the public in terms of: not speeding; helmet and seat-belt use; being seen; not drinking and driving; not using a mobile while driving; and not using drugs. In high-income countries, many activities take place and facilities exist to encourage these important courses of action among members of the public. However, these activities are not completely successful in most LMICs and in the EMR region. For example, a previous study in EMR countries, indicated that the major obstacles to visibility of the pedestrians on road were: a lack of knowledge regarding the importance of visibility; a negative attitude to using reflective materials; and a lack of availability of reflective materials in the country. ^44^ All these hindered visibility in the road system. The same situation can be found in most EMR countries; where there is lack of attention by government to providing such important measures. In a system approach, for example, Vision Zero, if the roads and motor vehicle safety cannot improve much, more emphasis should be put on reducing speed and vice versa.^2,13^ Accordingly, a comprehensive approach to speed management should take place in the EMR region. In many LMICs, the concept of speed management has not yet been accepted.^2^ It is important to note that this approach and its principles can be applied to any country regardless of the road transport system, and even at any stage of a country’s development. None of the EMR countries has passed the legislation or has such a vision in relation to RTI prevention. It is suggested that such a vision should be included as an indicator in future publication of Global Status Report on RTI prevention.

Compare to previous published article on Global Status Report in 2011, Somalia now has reported in new report in 2015 for these measures. Moreover, unlike other EMR countries that have fatal RTI reduction, Kuwait, Oman and Iraqi had more fatal RTIs. It is important to note that it may related to better registration and establishment of road traffic injury surveillance system, which can result more registration of fatal RTI. Moreover, focusing on child restraint law and its level of enforcement, passing law are increased from two countries to five. Mandatory seat-belt laws for both front-and rear seat occupants, child restrain as well as mandatory helmet laws for both drivers and passengers have increased from two countries to five countries. 

**Strengths and limitations**

Following the first previous published article in this line, this is the next article of secondary data analysis on Global Status Report of World Health Organization in 2015. The article follows comparison of policies in EMR that may be used as benchmark in both EMR and the other WHO regions for better interventions and policies. Since these data are governmental report, we can rely on the accuracy of the data. However, the base of data are registry base that may suffered from underestimation. In order to overcome that, it is important to note that WHO also has its own estimation. The authors also used correction factor to clarify the level of underreporting. 

## Conclusion

Although there have been some achievements on road safety in EMR compared to the 2009 WHO report, much effort is needed to optimize the enforcement of existing road safety laws. A maximum urban speed limit should be introduced in many region states. A system approach should be used and sufficient attention should be paid to the needs of vulnerable road users including pedestrians, cyclists and motorcyclists, who together make up about 50% of EMR road traffic deaths. Lessons can be learnt from the successful use of a system approach as well as the implementation of Vision Zero in some high-income countries which has led to successful progress in RTI reduction. The inclusion of this measure in the Global Status Report on Road Safety may improve RTI measures in EMR countries.

**List of abbreviations**

**EMR: **Eastern Mediterranean Region

**GNI: **Growth National Income

**HICs: **High-Income Countries

**LMICs: **Low- and Middle-Income Countries

**RTI: **Road Traffic Injury

**WHO: **World Health Organization

**Acknowledgements**

The authors would like to acknowledge the contributions of Safety Promotion and Injury Prevention Research Center in Shahid Beheshti University of Medical Sciences for their contribution of this study.
